# A Case of Polymyxin b-Immobilized Fiber Column Treatment for Rapidly Progressive Interstitial Pneumonia Associated with Clinically Amyopathic Dermatomyositis

**DOI:** 10.1155/2013/750275

**Published:** 2013-07-28

**Authors:** Oh Sasaki, Makoto Dohi, Hiroaki Harada, Mitsuru Imamura, Yumi Tsuchida, Kensuke Yamaguchi, Toshihiko Komai, Kazuhiko Yamamoto

**Affiliations:** Department of Allergy and Rheumatology, Graduate School of Medicine, University of Tokyo, Hongo 7-3-1, Bunkyo-ku, Tokyo 113-0033, Japan

## Abstract

We report a case of rapidly progressive interstitial pneumonia associated with clinically amyopathic dermatomyositis who responded to single course of polymyxin b-immobilized fiber column treatment. Initial treatment with pulsed corticosteroids and cyclophosphamide, intravenous immunoglobulin, and cyclosporine seemed to suppress the activity of interstitial lung disease temporarily, but signs of relapse were detected such as elevation of serum KL-6 level and progressing pulmonary shadows in chest computed tomography scan. After polymyxin b-immobilized fiber column treatment, the areas of pulmonary shadows drastically decreased. Gradually, arterial partial pressure of oxygen/fraction of inspired oxygen (PaO_2_/FiO_2_) ratio recovered, and serum ferritin level and KL-6 level decreased. These findings indicate that polymyxin b-immobilized fiber column treatment could be promising in combination with conventional therapy for rapidly progressive interstitial pneumonia associated with clinically amyopathic dermatomyositis, especially at the early phase of relapse.

## 1. Introduction

Dermatomyositis (DM) is an idiopathic connective tissue disease which is characterized by inflammation of the muscles and the skin. It is also a systemic disease involving the joints, esophagus, lungs, and heart. Clinically amyopathic dermatomyositis (C-ADM) is characterized by typical cutaneous manifestations and minor involvement of skeletal muscles [1]. It is well known that the interstitial lung disease (ILD) associated with C-ADM often presents with rapid onset and progression [2] and that the type of ILD is resistant to conventional therapy and has a poor prognosis especially in East Asia [3, 4].

It has been reported that direct hemoperfusion with polymyxin b-immobilized fiber column (PMX) effectively removed circulating endotoxin both *in vitro* and *in vivo* studies [5]. It was initially developed as a treatment for sepsis, and it has also revealed favorable effects on arterial partial pressure of oxygen/fraction of inspired oxygen (PaO_2_/FiO_2_) ratio in acute respiratory failure [6]. Here, we describe a patient of rapidly progressive interstitial pneumonia associated with C-ADM whose relapse after intensive conventional therapy was successfully treated with PMX hemoperfusion.

## 2. Case Report

A 37-year-old man presented with skin rash for the last two months. He also had progressing dyspnea for the last two weeks and visited a hospital to consult with a physician. His oxygen saturation (SpO_2_) was as low as 91% at room air in resting state, and he was admitted to the hospital. He had skin rashes in the face and on the hands. Chest computed tomography (CT) scan revealed nonsegmental pulmonary infiltrate and grand-glass opacity mainly distributed in the subpleural areas of the lower lobes. In addition, there were some legions of consolidation with air bronchogram in the peripheral lung, which implied traction bronchiectasis. The bronchoalveolar lavage fluid (BALF) analysis showed increases in total cells (2.25 × 10^5^/mL) and lymphocyte fraction (64.5%). Culture of BALF was negative. In transbronchial lung biopsy samples, lymphocytes and macrophages was infiltrated, and masson body-like fibrotic foci was formed.

He was suspected of rapidly progressing interstitial lung disease with dermatomyositis and transferred to our hospital. He had Gottron's papules, mechanic's hands, and spotted rashes between eyebrows, but he did not have heliotrope rash or other rashes on joints or body trunk. Manual muscle testing revealed no muscle weakness both in proximal and distal muscles. Fine crackles were audible in the bilateral lower lung fields. Laboratory findings on admission revealed a white blood cell count of 4,200/mm^3^ with 76.1% neutrophils C-reactive protein was slightly elevated (3.21 mg/dL [standard value; 0–0.4 mg/dL]). His serum lactate dehydrogenase (LDH), KL-6, ferritin, aldolase, and D-Dimer levels were elevated (586 IU/L [119–229 IU/L]), 1835 U/mL [<500 U/mL], 842 ng/mL [20–280 ng/mL], 14.1 U/L [2.7–7.5 U/L], and 12.4 *μ*g/mL [<1.0 *μ*g/mL], resp.), but creatine kinase (CK) was not elevated (201 U/L [55-210 U/L]). He did not have antinuclear antibody, anti-Jo-1 antibody, or other anti-aminoacyl-tRNA synthetase antibodies. Immunoprecipitation with the patient's serum revealed that he had a 140 kD protein band, which is supposed to be C-ADM-140/MDA5 antibody; however, further determination was not available in our case. Arterial blood gas test at O_2_ inhalation of 3 L/min with nasal cannula revealed decreased levels of PaO_2_ (68.7 Torr) and PaCO_2_ (31.7 Torr). Plasma b-type natriuretic peptide (BNP) was <4.0 pg/mL [<18.4 pg/mL], and ultrasound cardiography revealed that his cardiac function was normal. Skin biopsy was performed, and the findings were compatible with dermatomyositis. Electromyogram or magnetic resonance imaging of thigh muscles was omitted because respiratory failure was progressing and the treatment was made the highest priority. There were no signs of malignancy so far as we could check.

He was diagnosed as clinically amyopathic dermatomyositis (C-ADM) with rapidly progressing interstitial pneumonia and treated with pulsed corticosteroids (methylprednisolone 1 g/day, days 0–3, 16–18) followed by high dose corticosteroids, intravenous cyclophosphamide (IVCY; 500 mg/m^2^, day 3, 22; 750 mg/m^2^, day 41), intravenous immunoglobulin (20 g/day, days 6–10, 48–52), and cyclosporine-A (CSA; 200 mg/day, days 0–5; 250–300 mg/day, days 6-). Anticoagulation treatment with heparin followed by warfarin was also used (days 0-). In response to these initial treatments, consolidations and grand-glass opacities decreased in chest CT scans (Figure 2, day 7). Oxygen demand decreased from 4 L/min to 1-2 L/min to maintain SpO_2_ level at 90–95% in resting state. However, serum ferritin kept increasing and began to decrease two weeks afterwards.

**Figure 1:**
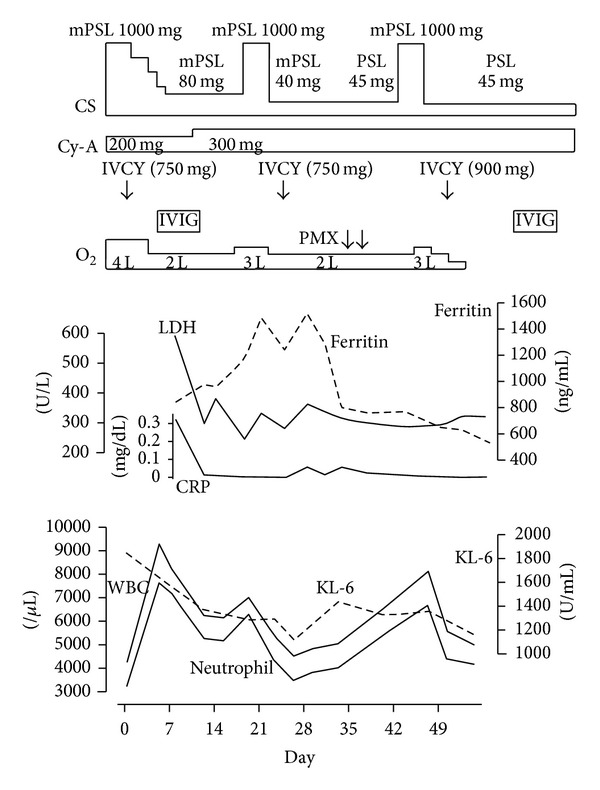
Time course of medical treatments and changes in the lab data before and after treatment. CS: corticosteroids; mPSL: intravenous methylprednisolone including pulse therapy; PSL: oral prednisolone; Cy-A: cyclosporine A; IVCY: intravenous cyclophosphamide therapy; IVIG: intravenous immunoglobulin (20 g/d for 5 days); PMX: polymyxin B-immobilized fiber column hemoperfusion; O_2_: oxygen supplied with nasal cannula; LDH: serum lactate dehydrogenase; ferritin: serum ferritin level; CRP: serum C-reactive protein; KL-6: serum sialylated carbohydrate antigen KL-6 level; WBC: white blood cell count; neutrophil: neutrophil count.

**Figure 2:**
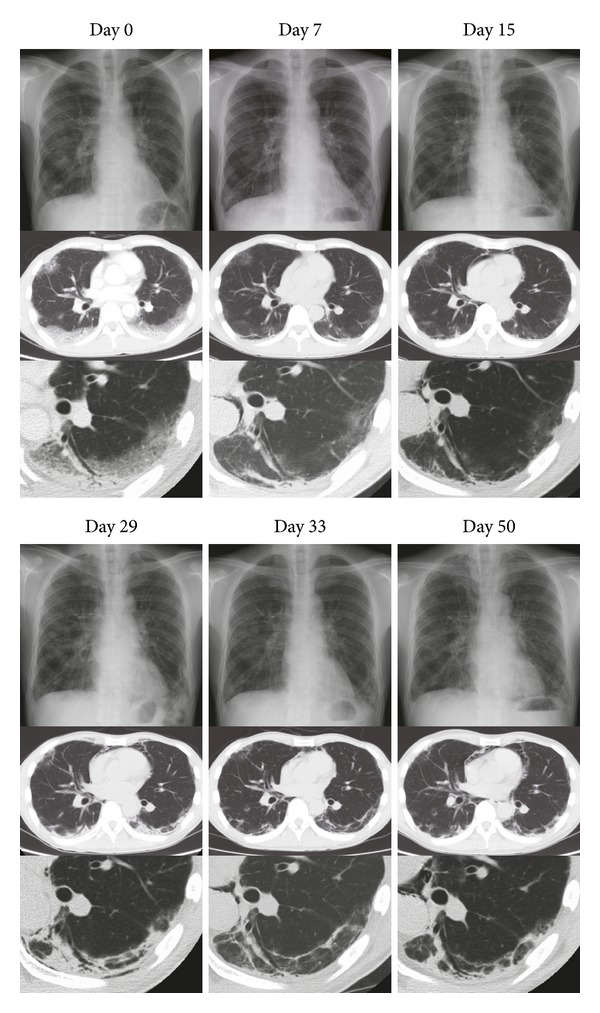
Chest X-rays and computed tomography (CT) scans of the patient with clinically amyopathic dermatomyositis (C-ADM) before and after polymyxin b-immobilized fiber column hemoperfusion (PMX) treatment. Day 0, on admission. Day 7, improvement after initial treatment. Day 15, before relapse. Day 29, on relapse and before PMX treatment. Day 33, improvement after PMX treatment (on days 30 and 31). Day 50, twenty days after PMX treatment.

On day 29, chest CT scans revealed progressing interstitial shadows again (Figure 2). CK was not elevated (130 U/L). Plasma BNP was slightly elevated (26.9 pg/mL), but there were no signs of heart failure. Although his oxygen demand or respiratory symptoms had not changed significantly, we thought that his interstitial lung disease was relapsing. Since a relapse after initial treatment of rapidly progressive ILD is often fatal, we decided to conduct intensive care in this early phase of the relapse, thus applied hemoperfusion treatment in this moment. On days 30 and 31, he underwent single course of PMX treatment, which was carried out at a flow rate of 80–100 mL/min for 12 h, twice at an interval of 24 h. Nafamostat mesilate (Torii Co., Ltd, Tokyo, Japan) was used for anticoagulation. Following the hemoperfusion, the findings detected by chest X-ray and CT scans improved remarkably, and the serum ferritin kept decreasing. Oxygen demand improved gradually, reflected in the increase in arterial partial pressure of oxygen/fraction of inspired oxygen (PaO_2_/FiO_2_) ratio (from 215 to 313), whereas serum concentration of LDH and KL-6 still remained at high level (Figure 1). The dosage of oral corticosteroid was tapered gradually, and he was discharged with a prescription of 30 mg of prednisolone and 250 mg of cyclosporine. He was treated with intravenous cyclophosphamide repeatedly. There has been no recurrence in him for 6 months after the discharge.

## 3. Discussion

We report a case of rapidly progressive ILD with C-ADM, whose relapse in spite of intensive therapy was successfully prevented by PMX hemoperfusion. We think that PMX is a potent therapeutic option in combination with the conventional treatment, especially in the early phase of the relapse of the ILD with C-ADM.

Acute/subacute interstitial lung disease in patients with polymyositis/dermatomyositis (PM/DM) is frequently fatal within months despite high dose prednisolone (PSL) therapy [7]. Acute/subacute ILD is more common in patients with C-ADM, which results in higher mortality rate in these patients [8]. It is often resistant to conventional therapy and has poor prognosis especially in East Asia [3, 4]. It was reported that HRCT patterns differed between anti-C-ADM-140 positive and negative patients in DM; lower lobes consolidation or ground-glass attenuation (GGA) pattern (50.0%) and random GGA pattern (33.3%) were the predominant patterns, and intralobular reticular opacities were absent in anti-C-ADM-140 positive cases, while lower lobes reticulation pattern (69.2%) was frequently seen in anti-C-ADM-140 negative cases [9]. NSIP and DAD were seen in histological examinations of acute/subacute ILD with C-ADM [10]. Furthermore, C-ADM-associated interstitial pneumonia takes an aggressive course even when the radiological and histological features are consistent with NSIP [11]. In the present case, the radiological features were mostly consistent with lower lobes consolidation or GGA pattern, and there was traction bronchiectasis on admission, which is a predictor of rapid progression and poor prognosis of ILD [12]; therefore he received intensive initial treatment with pulsed corticosteroids and IVCY, intravenous immunoglobulin, oral CSA, and warfarin.

It was reported that combination therapy with pulsed corticosteroids, CSA, and IVCY was promising for acute/subacute ILD with DM [7]. It was reported that early intervention with CSA in combination with corticosteroid was effective, with tightly monitoring 2 h postdose blood concentration (C2) of CSA [4, 13]. In this case, we also used CSA as an initial treatment and monitored trough as well as C2 level of CSA. There are reports that intravenous immunoglobulin is effective for ILD with DM/PM [14, 15], and we also applied it twice with an interval of a month.

Although pulmonary coagulopathy is suspected to be involved in the pathogenesis of ILD, effectiveness of anticoagulation therapy for ILD is controversial [16–18]. As a maintenance therapy for idiopathic pulmonary fibrosis (IPF), warfarin had negative effect [16]. On the other hand, anticoagulation is known to be beneficial in acute exacerbation of various ILDs including IPF and C-ADM [17, 18]. In our case, the patient presented with elevated plasma D-Dimer on admission, which might have indicated thrombus formation in pulmonary microcirculation, and we decided to begin anticoagulant therapy as an acute phase treatment.

In the present case, the intensive initial treatment with pulsed corticosteroids and cyclophosphamide twice, respectively, intravenous immunoglobulin once, and oral cyclosporine and warfarin succeeded in the induction of the remission to the rapidly progressing ILD with C-ADM. However, relapse was suspected soon after the initial treatment as progressing interstitial shadows without any signs of heart dysfunction. Although CT findings at the time of suspected relapse were not so prominent, we judged thatitwas a critical time point to decide good or poor prognosis; thus we conducted polymyxin b-immobilized fiber column (PMX) treatment.

PMX treatment was initially developed as a treatment for sepsis by removing circulating endotoxin. It has also revealed favorable effects on (PaO_2_/FiO_2_) ratio in acute respiratory failure [6]. The number of reports is limited, but it was also effective for rapidly progressing ILD [19–21]. It has been reported that PMX treatment was also effective for ILD with C-ADM in combination with conventional treatment [22–24]. Although there remains the possibility that the other immunosuppressants finally become effective, it seems that PMX played the major role in this case, since the progressing pulmonary infiltrates quickly cleared just after the PMX treatment.

However, the mechanism by which PMX improves pulmonary oxygenation in acute exacerbation of ILD (AE-ILD) has not been elucidated [25]. The removal of plasma endotoxin does not have a major role, because serum levels of endotoxin were within the normal range in previously reported cases of AE-ILD [19, 22, 25–27]. PMX treatment was known to reduce metalloproteinase-9 and tissue inhibitor of metalloproteinase-1 [28], neutrophil reactive oxygen species [29], neutrophil elastase, and interleukin-8 [30]. These mediators cause lung injury and increase vascular permeability as well as the intrapulmonary shunt ratio. These mediators are known to be released from neutrophils and monocytes. It has been shown that the polymyxin b-immobilized fiber column itself traps activated blood neutrophils and monocytes, which may be involved in the reduction of releasing mediators [31–33]. In our case, blood neutrophil count and monocyte count were not reduced after PMX treatment. The mechanism by which PMX treatment improved the ILD should be studied further. A long perfusion duration (12 h) of PMX had better prognosis than short course (<6 h) for AE-ILD [19, 25], and we also applied 12 h for a hemoperfusion duration.

It has been reported that serum ferritin correlates with the activity of the ILD with C-ADM [34]. In our case, serum ferritin kept increasing for about a week after initiating intensive immunosuppressive therapies and began to decrease afterwards. There seemed to be a time lag of approximately two weeks between the decline of disease activity and decrease of the serum ferritin level.

KL-6 is a mucin-like glycoprotein expressed on type II pneumocytes. Serum KL-6 level has diagnostic and prognostic significance in ILD or acute respiratory distress syndrome [35, 36]. It has better diagnostic accuracy of ILD in comparison to SP-A, SP-D, and MCP-1 [37, 38]. Serum KL-6 is known to be elevated even 7–10 days after successful treatment [39, 40], which is compatible with our case.

Anticlinically amyopathic dermatomyositis- (C-ADM-) 140/MDA5 autoantibodies have been known to be a specific marker for C-ADM and strongly associated with rapidly progressive ILD with C-ADM [41]. Furthermore, their quantification may be useful for monitoring ILD activity [42].

In summary, we report a case of rapidly progressive interstitial pneumonia associated with C-ADM, whose relapse after intensive initial treatment was successfully prevented with single course of PMX hemoperfusion. PMX may be a potent therapeutic option in combination with conventional therapies for ILD associated with C-ADM, especially in the early phase of the relapse.
